# ASSOCIATION OF EVERYDAY LIFE INFORMATION ACQUISITION METHODS AND DEPRESSION AMONG US OLDER ADULTS

**DOI:** 10.1093/geroni/igad104.2720

**Published:** 2023-12-21

**Authors:** Wuyi Dong, Yan-Jhu Su, Andrew Alberth

**Affiliations:** University of Massachusetts Boston, Boston, Massachusetts, United States; University of Massachusetts Boston, Boston, Massachusetts, United States; University of Massachusetts Boston, Boston, Massachusetts, United States

## Abstract

Everyday life information acquisition refers to the incidental ways people obtain information through daily activities such as reading, watching television, or communicating with friends rather than through intentional or purposive information seeking behaviors. It has been identified as a fundamental way to obtain informational resources and is a potential means to promote social connections and engagement among older adults. Research has shown that social engagement and connections are protective factors of depression for older adults. However, little research has explored how information seeking behaviors effect risks of depression among older adults. To fill this gap in the literature and provide insights into the mechanism of information access impacts the mental health of older adults, this study examines the relationships between different ways for obtaining daily information (reading, using computer, and watching television) and depression among older adults in United States. The data retrieved from 2018’s Health and Retirement Study (HRS). We multiple linear regression analyses on HRS data including adults age 50 years and older (n=3,179). After adjusting for age, gender, race, marital status, education, and chronic diseases, all three different ways to obtaining daily information (reading, using computer, and watching television) were negatively associated with depression (β=-0.19, p< 0.001; β=-0.15, p=0.002; β=-0.15, p< 0.001, respectively). Findings suggest that the association between everyday life information acquisition and depression among older adults varies depending on the specific information acquisition methods used. These findings highlight the importance of implementing targeted interventions to promote effective information access and enhance social inclusion among older adults.

